# COVID-19: a closer look at the pathology in two autopsied cases. Is the pericyte at the center of the pathological process in COVID-19?

**DOI:** 10.4322/acr.2021.262

**Published:** 2021-05-06

**Authors:** Hubert Daisley, Arlene Rampersad, Martina Daisley, Amit Ramdin, Oneka Acco, Farhaana Narinesingh, Ornella Humphrey

**Affiliations:** 1 General Hospital San Fernando, Department of Pathology, Trinidad, West Indies; 2 Scarborough General Hospital, Department of Accident and Emergency, Tobago, West Indies; 3 University of the West Indies, St. Augustine, Trinidad, West Indies

**Keywords:** Microcirculation, Pericytes, Fibrin, SARS virus

## Abstract

We performed autopsies on two cases of COVID-19. The microcirculations of all organs were the site of the pathological findings. Thrombotic microangiopathy was found in the brain and also the kidneys. Vasculitis was also a feature of the autopsy findings, together with portal triaditis of the liver. The major pathological findings in both cases were fibrin deposits. Within the lung, the fibrin deposits were observed in the alveolar microcirculation in sub-endothelial locations of capillaries, arterioles, post capillary venules, and the adventitia of larger vessels. These fibrin deposits in the lungs occurred at the sites where pericytes are located in these vessels. The pericyte with its high concentration of ACE-2 receptors and its procoagulant state may represent one of the primary sites of action of SARS-CoV-2. A review of pericytes in health and disease is undertaken. COVID-19 is a disease of the microcirculation.

## INTRODUCTION

COVID-19 has affected the lives of everyone socially, economically or medically. At the time of writing, there has been thirty-eight million confirmed cases with over one million and eighty-five thousand deaths from COVID-19 globally.^1^ The pandemic continues unabated. SARS-CoV-2 the virus of the COVID-19 pandemic is attracted to ACE-2 receptor sites causing an endotheliopathy^2^ with the lungs having the main pathological findings.^3^ Dysfunction of the microcirculation^4^ occurs which leads to hypoxic injuries of tissues and organs throughout the body, causing morbidity and mortality in the elderly and those with co-morbidities such as diabetes mellitus, hypertension, chronic lung diseases, and the multi-system inflammatory syndrome in infants and young adults.^5^

Treatment is mainly preventative and symptomatic. Wearing a mask, washing of the hands and social distancing are the defense mechanisms used to prevent the spread of this respiratory virus. Immune therapy, antiviral drugs and ventilator assistance have been used in the fight against COVID-19, with measured success.^6^^,^^7^ A vaccine against SARS-CoV-2 is forthcoming.

Understanding the pathophysiology of COVID-19 is most important in designing therapy. A closer look at the structure and function of the microcirculation with focus on the endothelial cells, and more so the pericyte, sheds insights into the secrets of COVID-19.

## CASE REPORTS

The autopsies were done under the guidelines of the Ministry of Health in the Republic of Trinidad and Tobago. Tissues for examination were processed and stained with H&E and CD31 (endothelial marker). Nasopharyngeal swabs for Covid-19 in both cases were processed using Abbott Real-time SARS-CoV-2 assay polymerase chain reaction (PCR) with homogenous real-time fluorescent detection. Reference weights for organs stated in brackets were taken from Autopsy Pathology: A Manual and Atlas (3^rd^ edition) by Andrew Connolly et al.^8^

## CASE 1

### Case history

A 16-year-old male collapsed shortly after having refreshments and was taken to the Accident and Emergency department of the General Hospital, where cardiopulmonary resuscitation was instituted, but he died in spite of resuscitative attempts. He had no known medical condition or psychosocial problems. He attended secondary school, but because of the COVID-19 pandemic was tutoured at home via online video conferencing like all Secondary and Primary school children on the island. Neither he nor his family had been in contact with persons suffering with COVID-19.

An autopsy was compulsory.

### Autopsy presentation

The body was that of a well-nourished young male, 155 cm long, asthenic and of appearance consistent with the stated age. Lividity was on the back.

He had pink mucous membranes, was anicteric, and cyanosed at the nail beds. There was no edema, clubbing, lymphadenopathy, or abnormality of the skin and the musculoskeletal system.

The cranium was grossly unremarkable and also was the sagittal sinus. There were no extradural or subdural hematomas. There was vascular congestion of the meninges, and the vessels of the circle of Willis were structurally unremarkable. The brain weighed 1300 g (1440+/-30 g), was edematous and there was no other gross pathology of the brain even upon serial sectioning.

There were 30 mL of serous pericardial effusion. The coronaries were patent and originated from their respective ostia, and had no gross pathology. There was no thrombus of the atria. The heart valves circumferences were as follows: Mitral 9 cm (7.8+/-0.7 cm), Aortic 6.5 cm, (5.3+/- 0.4 cm), Tricuspid 10 cm, (9.9+/-0.9 cm) Pulmonary 5.4 cm (5.6+/-0.5cm). The left ventricle thickness was 1.0 cm, and the right ventricle 0.2 cm. There was no myocardial injury, present or remote. There was cardiomegaly 270 g (RR; 125-250 g).

The pulmonary arteries contained no thromboembolism. There were approximately 350 mL of serosanguinous pleural effusions in each pleural cavity. The larynx was structurally unremarkable, and there was hyperemia of the trachea. There was no foreign body within the tracheobronchial tree or tumor. The right lung weighed 770 g (455 g upper limit of normal), and the left lung 710 g (415 g upper limit of normal) and were markedly congested, edematous, and contained no area of consolidation.

There were no bite marks of the tongue; no chemical burns of the oropharynx, or esophagus. The stomach contained a clear, non-pungent fluid and there was no gastritis. The duodenum and the rest of the gastrointestinal tract were structurally unremarkable. There was free flow of bile. The liver weighed 1950 g (1523 g upper limit of normal), and had a focal area of sub-capsular hemorrhage, attributable to an injury obtained during resuscitative attempts. The rest of the liver was congested. The spleen weighed 200 g (RR; 150-200 g), was congested, and revealed no abnormality upon serial sectioning.

The kidneys each weighed 90 g (RR; right kidney 90-110 g, left kidney 98-112 g), had smooth cortical surfaces, and normal cortico-medullary junctions. The adrenals combined weight was 20 g (RR; 16-20 g), and were grossly unremarkable. The ureters and the urinary bladder were structurally unremarkable, with the latter containing 50 mL of amber-coloured urine.

The genitals were structurally unremarkable.

Nasopharyngeal swab testing for COVID 19 using PCR, performed after autopsy was positive.

### Histology

**Figure 1:**
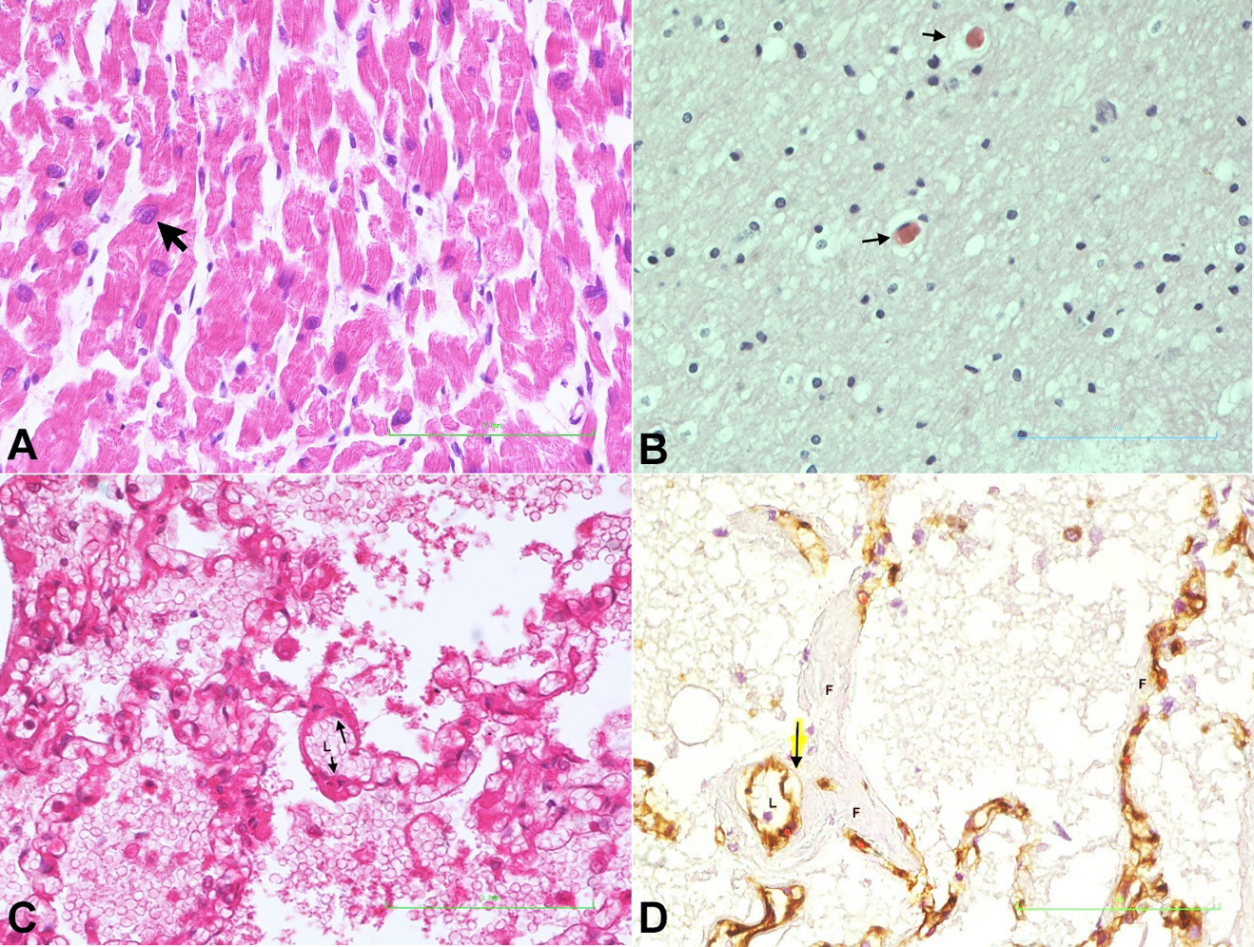
Photomicrographs of: **A –** Cardiomyocyte showing enlarged nucleus (arrow) (H&E x 400); **B –** Brain showing thrombotic microangiopathy (arrows) (H&E x 400); **C –** Alveolar-capillary section showing subendothelial fibrin deposits (arrows) lumen represented as L (H&E x 200); **D** – Pulmonary section showing unstained subendothelial fibrin deposits (F), brown-stained endothelial cells (arrow) with lumen free of thrombus (L) (CD31 stain x 400).

**Heart:** There was idiopathic cardiomegaly with enlarged myocyte nuclei, some showing bizarre forms (Figure 1A).

**Brain:** The brain showed thrombotic microangiopathy (Figure 1B). In addition, medium sized vessels were thrombosed and showed a vasculitis with a moderate infiltrate of lymphocytes in the immediate perivascular region.

**Lung:** There was hyaline membrane, pulmonary edema, pulmonary hemorrhage and areas of interstitial pneumonia. Fibrin microthrombi were evident within alveoli capillaries, arterioles, and post capillary venules. In many areas, the fibrin thrombi were within the wall of capillaries, arterioles and venules in the sub-endothelial locations, but not within the lumen of these vessels (Figure 11D). Fibrin deposits were also observed in the adventitia of the large pulmonary vessels. These sites of fibrin deposition correspond with the locations of the pericytes in the vascular system.

**Figure 2:**
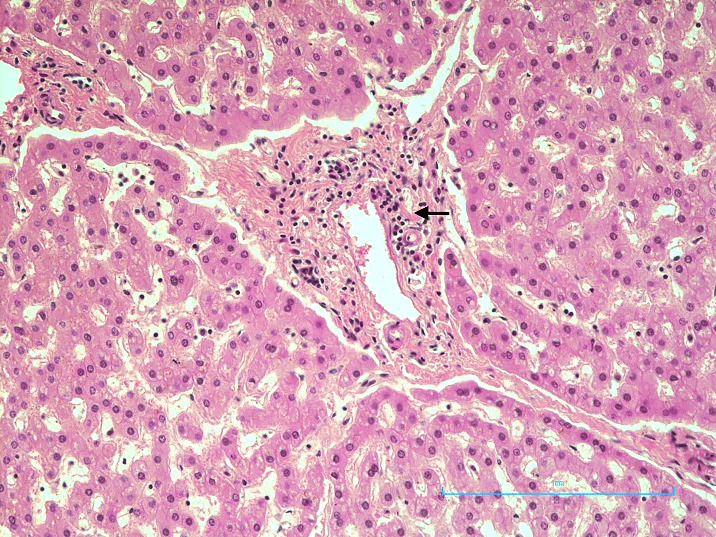
Photomicrograph of the liver showing portal triaditis (H&E x 200).

**Liver:** There was a moderate infiltrate of lymphocytes within the portal triad (Figure 2).

This COVID-19 case showed both the inflammatory and thrombotic responses to COVID-19 occurring in the lung and brain. This young male had idiopathic cardiomegaly and died in congestive cardiac failure, with superimposed COVID-19.

## CASE 2

### Case history

The deceased was an 84-year-old male with urinary retention due to benign prostatic hyperplasia and was managed with an indwelling Foley catheter. He had no other known medical illnesses. He was discovered dead at home in his bathroom. His family indicated that he was clinically well prior to his death. There was no history of diabetes mellitus, hypertension, recent travel, exposure to infectious persons, toxic agents, or any history of tobacco smoking or alcohol use. He dwelled in his home with his elderly wife and grandchildren.

A partial autopsy was performed.

### Autopsy presentation

The body was that of a well-preserved, elderly male, cyanosed at the nail beds, and with no external marks of violence or trauma. The mucous membranes were pale. A urinary catheter was in-situ.

The trachea and lower airways were hyperemic. There was no tumor or foreign bodies in the tracheobronchial tree. There were 50 mL and 30 mL of serous effusions in the right and left pleural cavities respectively. The left lung weighed 750 g (RR; 325-480 g) and the right lung, 860 g (RR; 360-570 g). They were congested and edematous. There was no hilar lymphadenopathy. There were areas of consolidation at the bases of all lobes of the right and left lungs. There was no evidence of pulmonary embolism.

The heart weighed 310 g (RR; 270-360 g). The valve circumferences and ventricular thicknesses were within normal limits.

The coronary arteries showed mild atherosclerotic disease with approximately 20% stenosis of the left anterior descending, circumflex and right coronary arteries.

The myocardium revealed no recent or old injuries.

The liver weighed 1100 g (RR; 1500-1800 g) and spleen 150 g (RR; 90+/-40 g), both appeared congested with no other macroscopic evidence of abnormality.

Both kidneys weighed 130 g respectively (RR; 313 g combined weight) and showed an ill definition of the cortico-medullary junctions and chronic pyelonephritis. The prostate was enlarged, with a prominent middle lobe, and the urinary bladder contained cloudy urine.

There was no injury to the scalp or the underlying skull bones. The brain was not examined.

COVID-19 Test on nasopharyngeal swab, done at autopsy using PCR was positive.

### Histology

**Figure 3:**
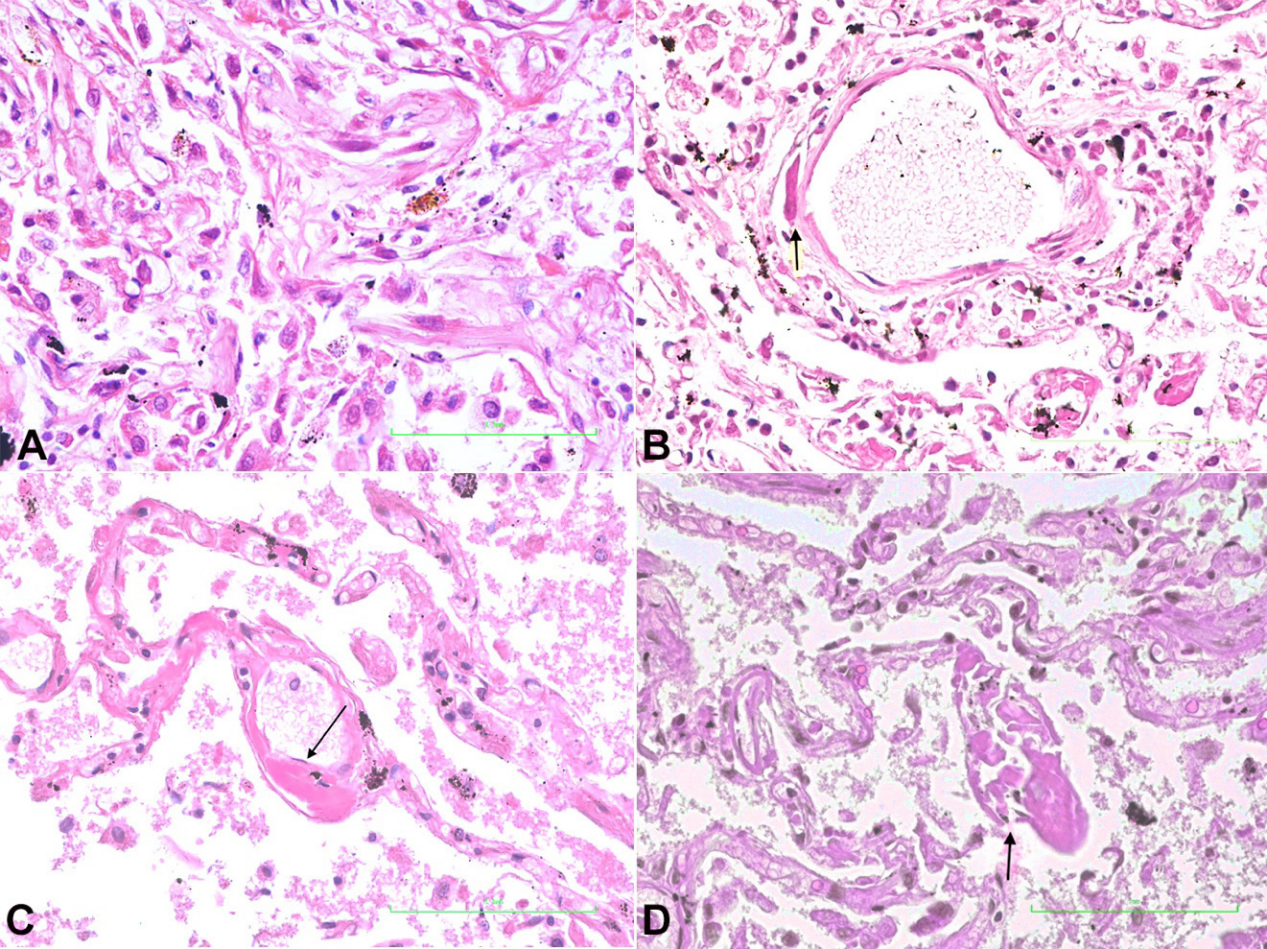
Photomicrographs of the lung. **A –** Pulmonary section showing interstitial pneumonia (H&E, x 400); **B –** Pulmonary section showing fibrin deposit (arrow) within the wall of a venule (H&E, x 400); **C –** Pulmonary section showing subendothelial deposit in a capillary (arrow) (H&E, x 400); **D –** Pulmonary section showing fibrin deposit in subendothelial location and subsequent destruction of the capillary wall (arrow) (H&E, x 400).

**Lungs:** This showed interstitial pneumonia. (Figure 3A) Fibrin deposits were distributed throughout the alveoli microcirculation, within the subendothelial locations of capillaries, arterioles, venules, and the adventitia of large pulmonary vessels (Figure 33C, 444D).

**Figure 4:**
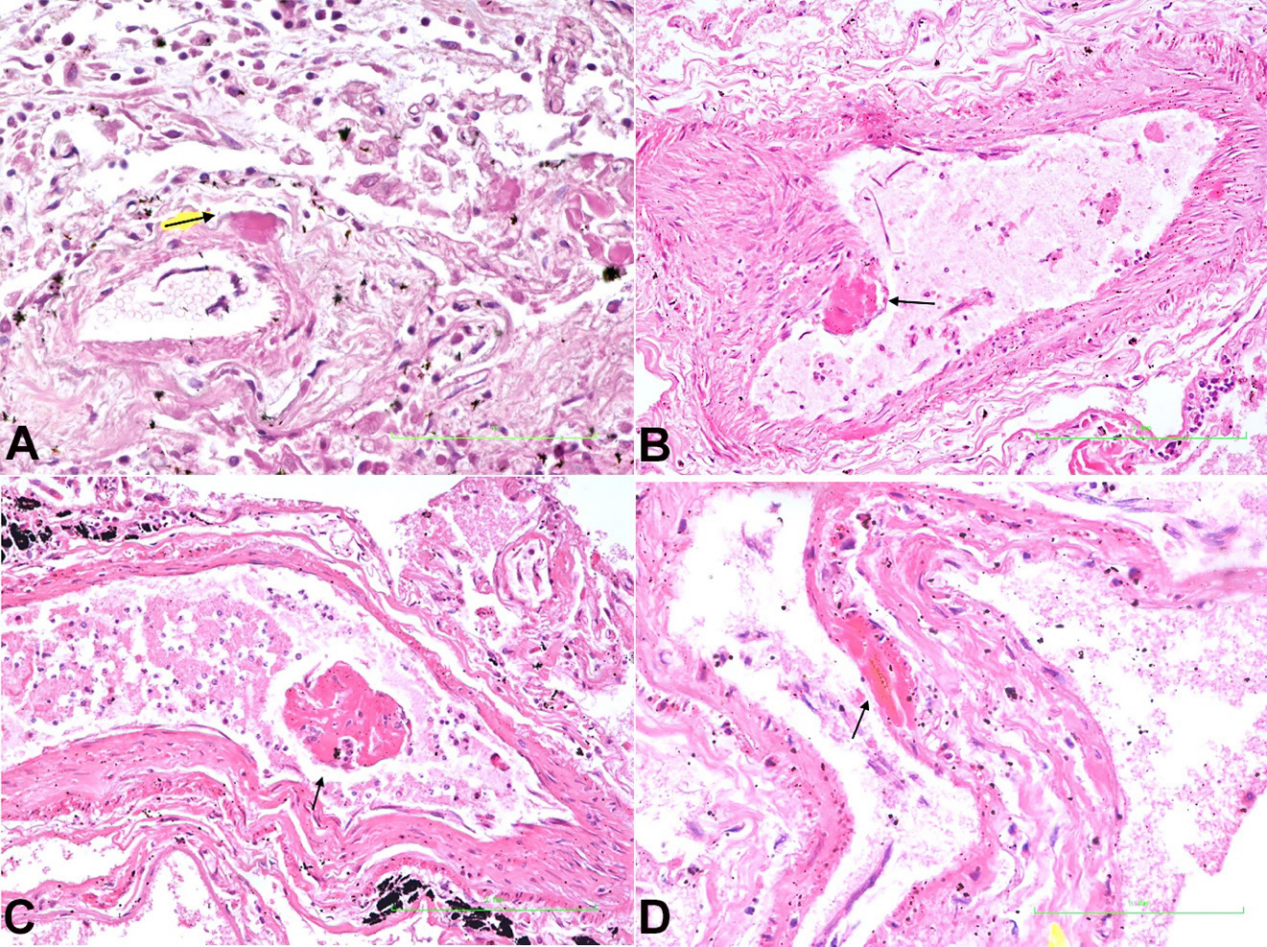
Photomicrographs of the lung. **A –** Pulmonary arteriole showing fibrin deposit in the adventitia (arrow) (H&E x 400); **B –** Pulmonary artery showing fibrin deposit underlying endothelial layer (arrow) (H&E x 400); **C –** Detached thrombus (arrow) in pulmonary vein (H&E x 400); **D –** Pulmonary post-capillary venule showing fibrin deposit underlying endothelial layer (arrow) (H&E x 400).

**Heart:** This showed focal ischemic changes globally.

**Kidney:** There were microthrombi within glomerular tuft and interlobular arteries.

**Liver:** There was portal triaditis.

This elderly male died from COVID-19 with diffuse fibrin deposits within the alveolar microcirculation and the adventitia of the larger pulmonary vessels. The resultant hypoxia would have contributed to multiple organ failure.

## DISCUSSION

The microcirculation is the terminal vascular network of the systemic circulation consisting of arterioles, capillary beds and post capillary venules. In this region, oxygen is transferred from red blood cells to tissues and organs. This facilitates respiration at the cellular and tissue level. Hypoxic injury occurs to tissues and organs if the oxygen delivery is interrupted in these micro-vessels. In COVID-19, interruption of oxygen delivery to tissues and organs occurs as a result of fibrin deposition within the microcirculation, thereby exerting its pathological effects.^9^

The pulmonary microcirculation resides within the alveolus. Here, the alveolar-capillary endothelial cells, contribute to and share a common basement membrane with type I and type II epithelial cells. The type I epithelial cells pave the alveolus, and is the site of gas exchange. In contrast, type II epithelial cells manufacture surfactant, reducing the surface tension within the alveolus to allow respiration at atmospheric pressure. They also divide to produce the type I cells.

Cross-talks between epithelial and endothelial cells occur therefore injury to epithelial cells can result in injury to endothelial cells. SARS-CoV-2 is a respiratory virus, which initially affects the respiratory tract but causes its major pathological effects in the alveolar microcirculation.^3^

The intima of the capillary is composed of a single layer of endothelial cells resting on a basement membrane. This forms the skeleton of the capillaries. Hidden on the abluminal side of the endothelial cell is the pericyte, with a complex network of matrix; which adheres to and encircles the luminal endothelial cell through peg-and-socket junctions, gap junctions, and adhesion plaques; and is embedded in the basement membrane. The matrix contains micro-particles, which are secreted by the pericytes themselves by exocytosis. The gap junctions allow the pericytes to communicate with the endothelial cells by exchanging of ions and small molecules. The peg-and-socket contacts enable pericytes to penetrate through the basal lamina and make contact with other cells and nearby vessels.^10^^-^^12^ One pericyte with its complex finger-like matrix can make contact with multiple endothelial cells. The pericyte contributes to the basement membrane, which is shared with the endothelial cell.

Pericytes are a heterogeneous population of cells. There are three categories of pericytes; ensheathing pericytes, which are found on precapillary arterioles; mesh pericytes; and thin stranded pericytes.^11^^,^^13^^,^^14^ These pericytes are found in the abluminal regions of the intima in arterioles, venules, capillaries, and in the adventitia of larger vessels.^15^

Pericytes play different roles in the microvasculature stability, barrier function and perfusion. They are involved in the preservation of vascular rheology and homeostasis, including regulation of blood flow, angiogenesis, structural stabilization of the vasculature, and vascular permeability.^16^^-^^18^

The endothelial cells, the abluminal pericyte and the basement membrane, are collectively called the endothelium, and any injury/dysfunction of this unit is often referred to as endotheliopathy. The endothelium has important roles in homeostasis, maintaining the balance between prothrombotic and antithrombotic states. The endothelial cells express thrombomodulin, which has anticoagulant properties keeping the blood in a fluid state. On the other hand, the pericytes are procoagulant^19^ and have a high concentration of Tissue Factor (TF), which is expressed within its cytoplasm, and as microparticles in its matrix/basement membrane.

In health, minor injuries to the microcirculation cause plasma to leak through gap junctions interacting with TF of the pericytes. Microthrombosis takes places as a result of the interaction. This microthrombotic event is an innate immune response to microvascular injury, which is intended to restore the integrity of the microcirculation. Hemostasis takes place with thrombomodulin, and fibrinolytic modulation in physiological states. However, pathological thrombosis of the microcirculation occurs in COVID-19,^19^ not only because of passive plasma flow and interaction with subluminal pericytes, but in addition, to adhesion of SARS-CoV-2 virus to ACE-2 receptor sites, thereby causing cytopathic effects on pericytes.

Other factors play a role in homeostasis. The major prothrombotic factors are TF; von Willebrand factor; protease-activated receptor; Thromboxane A2; von Willebrand platelet activating factor; plasminogen activator inhibitor-1; and adhesive molecules such as P-selectin, E-selectin, platelet adhesion molecule-1, and vascular cell adhesion molecule-1. These factors are counteracted by anticoagulants such as protein C, tissue factor inhibitor, thrombomodulin, heparin, nitric oxide synthase, profibrinolytic factors, anti-platelet factors, prostacyclin, and type 1 motif (ADAMTS13).^20^^,^^21^ Hence, there are a complex and heterogeneous admixture of plasma and cellular constituents that play an active role in homeostasis. The interplay amongst blood flow, the compositions of blood and vessel wall components are necessary to understand the operant pathophysiology of thrombotic and bleeding disorder and the maintenance of homeostasis.^21^

In sepsis and ischemia, inflammatory mediators are released from platelets and other inflammatory cells causing injury to the microcirculation. The endothelial cells contract and widen gap junctions, rendering the pericytes to mediate the leucocytes diapedesis through these gaps and endothelial cell junctions. Pericyte gap enlargement occurs under these conditions facilitating the flow of plasma and recruitment of more leucocytes in the injured region.^17^^,^^22^^-^^24^ Macrophage migration inhibition factor produced by macrophages, and released in the alveolar space, also plays a central role in the transmigration of neutrophils at sites of injury in the microcirculation.

Pericytes control the microvascular modeling and proliferative state of the endothelium and base membrane in angiogenesis.^17^^,^^25^^-^^27^

Notwithstanding the foregoing, the pericytes have been an overlooked player in the pathobiology of the disease. In fact, they play a central role in the micro-thrombosis, diapedesis of leucocytes, and angiogenesis after the injury to the microcirculation. All these properties of pericytes are manifested in COVID-19 disease.

ACE-2 receptor sites are the targets for the SARS-CoV-2 virus. These receptor sites are located on nasal and oral mucosae, nasopharyngeal cells, enterocytes, type I and type II epithelial cells in the alveolus, and endothelial cells in the microcirculation.^28^ Pericytes also contain high concentrations of ACE-2 receptor sites.^29^

In the two cases in discussion, one died suddenly and the other was found dead at his residence. Neither had ventilator assistance nor was on pharmaceutical therapy. Hence, the pathologic findings seen in these two cases were as a direct result of SARS-CoV-2 infection and its cytopathic effects on the endothelial cell, the subluminal pericyte and its basement membrane.

The teenage patient died from a combination cerebral thrombosis, thrombotic microangiopathy and vasculitis. These pathological events have been described in COVID-19 patients.^30^^,^^31^ He also had idiopathic cardiomegaly, which was discovered at autopsy. Congestive cardiac failure also contributed to his demise. Heart failure is a noted complication in COVID-19.^32^ It is interesting to note that cardiomyocyte expression of ACE-2 receptor sites are low, but the highest expression of ACE-2 receptor sites are in cardiac pericytes,^33^^,^^34^ which may represent the target cardiac cells in SARS-CoV-2 infection. The pericytes’ injury due to SARS-CoV-2 cytopathic effect may result in capillary endothelial cells dysfunction, inducing microvascular dysfunction. Therefore, heart disease patients, if infected by the SARS-CoV-2, may have a higher risk of myocardial injury, cardiac arrhythmias, heart failure, and critically ill conditions.^32^^,^^34^^,^^35^

A moderate portal triaditis was also a feature seen in this patient. This might represent part of the inflammatory response that occurs to SARS-CoV-2 infecting pericytes in vessels in the portal tract. The portal triaditis might be responsible for the abnormal liver function seen in COVID-19.^36^

It was within the lung that major pathological changes were observed. Fibrin was deposited in the sub-endothelial locations of the microcirculation in alveolar capillaries, venules and arterioles and in the adventitia of larger vessels. Disruption of the microvasculature walls in the alveolus by fibrin deposits led to extension of fibrin within alveolar spaces (Figure 3D) and vessel lumen (Figure 4C). The sites within the microcirculation, where fibrin deposits occurred, are the locations of the pericytes. With this understanding, we concluded that the pericyte with its high concentration of ACE-2 receptor sites, the target for SARS-CoV-2, and its procoagulant state, is at the center of the vascular coagulopathy seen in the lung. Although the alveolar epithelial cells contain ACE-2 receptor sites, epithelial cell proliferation was not as marked as was seen in H1N1 flu. However, there was patchy interstitial pneumonia. COVID-19 seems predominantly to be affecting the microcirculation, with fibrin deposit being the pathological feature.^19^ This disruption and destruction of the microcirculation by fibrin deposits and its extension into the alveolar spaces causes decreased oxygen exchange, resultant hypoxia and respiratory failure. Inflammatory mediators released during this hypoxic state gain entrance to the systemic circulation, thereby initiating the multiple organ dysfunction syndrome.^20^^,^^37^

Ackerman et al.^38^ described pulmonary vascular endothelialitis, thrombosis and angiogenesis in COVID-19. These pathological findings are all caused by the vascular pericytes.^17^^,^^22^^,^^23^^,^^27^^,^^39^ They also reported that endothelial cells in the specimens from patients with COVID-19 showed “disruption of intercellular junctions, cell swelling, and a loss of contact with the basal membrane”. These latter findings would certainly involve pericytes through the disrupted intercellular junctions. Although the pathobiology of the pericytes was recognized in their report, the pericytes was omitted from the discussion.

Frantzeskakil et al.^40^ in their article entitled “Immunothrombosis in Acute Respiratory Distress syndrome: Crosstalk between inflammation and coagulation”, recognized the Imbalance between coagulation and inflammation as a predominant characteristic of ARDS, leading to extreme inflammatory response and “diffuse fibrin deposition in vascular capillary bed and alveoli”. They also attributed TF production to the vascular endothelium. They did not recognize that the inflammation, the fibrin deposits in the capillary bed, and alveolar spaces in ARDS were all part of the pathobiology of the pericytes. Both Ackerman et al.^38^ and Frantzeskaki et al.^40^ overlooked the role of pericytes in the pathobiology of the microcirculation.

COVID-19 is a disease that affects the microcirculation^3^^,^^41^^,^^42^ with the pericytes playing a central role in the pathobiology of the disease process. The pericytes with its procoagulant state, its expression of TF, and its high concentration of ACE-2 receptor sites are the target cells for SARS-CoV-2 infection.^33^ The cytopathic effect of SARS-CoV-2 on endothelial cells exposes the abluminal pericytes, through gap junctions, to constituents of plasma and the SARS-CoV-2. The resultant cytopathic effect of SARS-CoV-2 on exposed pericytes generates the pathology of thrombosis, inflammation, and angiogenesis seen in COVID-19.

## CONCLUSION

SARS-CoV-2 virus attaches to receptor sites on both endothelial cells and pericytes of the microcirculation. It is possible that SARS-CoV-2 attachment to ACE-2 receptor sites on pericytes may be responsible for the major pathology seen in COVID-19. The current literature and the two autopsy findings support the involvement of the pericyte in SARS-CoV-2 pathogenesis. Further work is required to clarify the role of the pericyte in SARS-CoV-2 pathogenesis, if COVID-19 is to be efficiently managed.

## References

[1] Worldometer (2020). Coronavirus..

[2] Iba T, Connors JM, Levy JH (2020). The coagulopathy, endotheliopathy, and vasculitis of COVID-19. Inflamm Res.

[3] Kaur S, Tripathi DM, Yadav A (2020). The enigma of endothelium in COVID-19. Front Physiol.

[4] Kasal DA, De Lorenzo A, Tibiriçá E (2020). COVID-19 and microvascular disease: pathophysiology of sars-cov-2 infection with focus on the renin-angiotensin system. Heart Lung Circ.

[5] Jiang L, Tang K, Levin M (2020). COVID-19 and multisystem inflammatory syndrome in children and adolescents. Lancet Infect Dis.

[6] National Institutes of Health (2020). Immune-based therapy under evaluation for treatment of COVID-19..

[7] National Institutes of Health (2020). Antiviral drugs that are under evaluation for the treatment of COVID-19..

[8] Connolly A, Finkbeiner W, Ursell P, Davis R (2016). Autopsy pathology: a manual and atlas..

[9] McFadyen JD, Stevens H, Peter K (2020). The emerging threat of (micro)thrombosis in COVID-19 and its therapeutic implications. Circ Res.

[10] Armulik A, Abramsson A, Betsholtz C (2005). Endothelial/pericyte interactions. Circ Res.

[11] Cuevas P, Gutierrez-Diaz JA, Reimers D, Dujovny M, Diaz FG, Ausman JI (1984). Pericyte endothelial gap junctions in human cerebral capillaries. Anat Embryol.

[12] Winkler EA, Bell RD, Zlokovic BV (2011). Central nervous system pericytes in health and disease. Nat Neurosci.

[13] Zhao H, Chappell JC (2019). Microvascular bioengineering: a focus on pericytes. J Biol Eng.

[14] Bonkowski D, Katyshev V, Balabanov RD, Borisov A, Dore-Duffy P (2011). The CNS microvascular pericyte: pericyte-astrocyte crosstalk in the regulation of tissue survival. Fluids Barriers CNS.

[15] Corselli M, Chen CW, Sun B, Yap S, Rubin JP, Péault B (2012). The tunica adventitia of human arteries and veins as a source of mesenchymal stem cells. Stem Cells Dev.

[16] Ferland-McCollough D, Slater S, Richard J, Reni C, Mangialardi G (2017). Pericytes, an overlooked player in vascular pathobiology. Pharmacol Ther.

[17] Zhang ZS, Zhou HN, He SS, Xue MY, Li T, Liu LM (2020). Research advances in pericyte function and their roles in diseases. Chin J Traumatol.

[18] Davis GE, Norden PR, Bowers SL (2015). Molecular control of capillary morphogenesis and maturation by recognition and remodeling of the extracellular matrix: functional roles of endothelial cells and pericytes in health and disease. Connect Tissue Res.

[19] Ahmed S, Zimba O, Gasparyan AY (2020). Thrombosis in Coronavirus disease 2019 (COVID-19) through the prism of Virchow’s triad. Clin Rheumatol.

[20] Kwaan HC (2011). Microvascular thrombosis: a serious and deadly pathologic process in multiple diseases. Semin Thromb Hemost.

[21] Wolberg AS, Aleman MM, Leiderman K, Machlus KR (2012). Procoagulant activity in hemostasis and thrombosis: virchow’s triad revisited. Anesth Analg.

[22] Rudziak P, Ellis CG, Kowalewska PM (2019). Role and molecular mechanisms of pericytes in regulation of leukocyte diapedesis in inflamed tissues. Mediators Inflamm.

[23] Proebstl D, Voisin MB, Woodfin A (2012). Pericytes support neutrophil subendothelial cell crawling and breaching of venular walls in vivo. J Exp Med.

[24] Gane J, Stockley R (2012). Mechanisms of neutrophil transmigration across the vascular endothelium in COPD. Thorax.

[25] Kutcher ME, Herman IM (2009). The pericyte: cellular regulator of microvascular blood flow. Microvasc Res.

[26] Walpole J, Mac Gabhann F, Peirce SM, Chappell JC (2017). Agent-based computational model of retinal angiogenesis simulates microvascular network morphology as a function of pericyte coverage. Microcirculation.

[27] Chang WG, Andrejecsk JW, Kluger MS, Saltzman WM, Pober JS (2013). Pericytes modulate endothelial sprouting. Cardiovasc Res.

[28] Hamming I, Timens W, Bulthuis ML, Lely AT, Navis G, van Goor H (2004). Tissue distribution of ACE2 protein, the functional receptor for SARS coronavirus: afirst step in understanding SARS pathogenesis. J Pathol.

[29] Huertas A, Montani D, Savale L (2020). Endothelial cell dysfunction: a major player in SARS-CoV-2 infection (COVID-19)?. Eur Respir J.

[30] Jaunmuktane Z, Mahadeva U, Green A (2020). Microvascular injury and hypoxic damage: emerging neuropathological signatures in COVID-19. Acta Neuropathol.

[31] Tavazzi G, Pellegrini C, Maurelli M (2020). Myocardial localization of coronavirus in COVID-19 cardiogenic shock. Eur J Heart Fail.

[32] Mehra MR, Ruschitzka F (2020). COVID-19 illness and heart failure: a missing link?. JACC Heart Fail.

[33] Chen L, Li X, Chen M, Feng Y, Xiong C (2020). The ACE2 expression in human heart indicates new potential mechanism of heart injury among patients infected with SARS-CoV-2. Cardiovasc Res.

[34] Stone E, Kiat H, McLachlan CS (2020). Atrial fibrillation in COVID-19: a review of possible mechanisms. FASEB J.

[35] Gupta A, Madhavan MV, Sehgal K (2020). Extrapulmonary manifestations of COVID-19. Nat Med.

[36] Feng G, Zheng KI, Yan QQ (2020). COVID-19 and liver dysfunction: current insights and emergent therapeutic strategies. J Clin Transl Hepatol.

[37] Robba C, Battaglini D, Pelosi P, Rocco RMP (2020). Multiple organ dysfunction in SARS-CoV-2: MODS-CoV-2. Expert Rev Respir Med.

[38] Ackermann M, Verleden SE, Kuehnel M (2020). Pulmonary vascular endothelialitis, thrombosis, and angiogenesis in COVID-19. N Engl J Med.

[39] Bouchard BA, Shatos MA, Tracy PB (1997). Human brain pericytes differentially regulate expression of procoagulant enzyme complexes comprising the extrinsic pathway of bloodcoagulation. Arterioscler Thromb Vasc Biol.

[40] Frantzeskaki F, Armaganidis A, Orfanos S (2017). Immunothrombosis in acute respiratory distress syndrome: cross talks between inflammation and coagulation. Respiration.

[41] Colantuoni A, Martini R, Caprari P (2020). COVID-19 sepsis and microcirculation dysfunction. Front Physiol.

[42] Martini R (2020). The compelling arguments for the need of microvascular investigation in COVID-19 critical patients. Clin Hemorheol Microcirc.

